# Safeguarding Experiences of People in Mental Distress, Police and Healthcare Practitioners: An Integrative Review

**DOI:** 10.1111/jpm.70014

**Published:** 2025-08-14

**Authors:** Inga Heyman, Catriona Kennedy, Aileen Grant, Andrew Wooff

**Affiliations:** ^1^ School of Health and Social Care Edinburgh Napier University Edinburgh UK; ^2^ School of Pharmacy, Applied Sciences and Public Health Robert Gordon University Aberdeen UK; ^3^ School of Nursing, Midwifery and Paramedic Practice Robert Gordon University Aberdeen UK; ^4^ School of Applied Sciences Edinburgh Napier University Edinburgh UK

**Keywords:** crime and mental health, multidisciplinary care, patient experience, psychiatric emergency nursing, systematic literature reviews

## Abstract

**Introduction:**

Globally, there is demand on police and emergency health services to respond to people in mental distress. Research at this intersect has focused on decriminalisation of people with severe mental health disorders, police custody care or interagency collaborative models. There is little understanding of the experiences of stakeholders where mental distress is not associated with a severe mental disorder or criminal offence.

**Aim:**

To determine current knowledge about safeguarding of people in mental distress supported by police and healthcare practitioners.

**Method:**

A rigorous integrative review with 10 databases was searched January 2002 to January 2024.

**Results:**

The search filtered 12,451 titles and abstracts with 41 full‐text articles appraised. Three overarching themes emerged: Safeguarding and care experiences of people in mental distress; intoxication, self‐harm and aggression; professional perspectives and responses to people in mental distress.

**Discussion:**

Experiences are varied. Whilst there is evidence of compassion, many reported negative experiences when self‐harm and intoxication are involved, inconsistent professional responses and gaps in emergency police and mental health systems.

**Implications for Practice:**

Unscheduled care and community mental health nurses have a vital role to play in identifying and influencing interdisciplinary gaps in out‐of‐hours emergency health and police processes to support people in mental distress to prevent repetitive distress cycles. This calls for an urgent re‐imagining of unscheduled care mental health pathways, Specifically, where practice gaps impact on people who are intoxicated yet do not require inpatient care.

**Relevance to Mental Health Nursing (for Peer Reviewers and Editors Only):**

People in mental distress (PiMD) who come to police attention often require an interdisciplinary response. Unscheduled care and community mental health nurses play a key role in this support. This integrative review suggests there are systems gaps and variety in mental health and policing practice for PiMD, particularly for those who are intoxicated and/or who do not need inpatient care. Some PiMD experience cyclical, and at times, undignified and unsafe care. These gaps should be addressed through service redesign and sharing of evidence across disciplines whilst listening to and responding to perspectives of those experiencing mental distress in our communities.

**Summary**

- What is known on the subject?
  ○ Police and emergency health services are involved with a large proportion of individuals in mental health crisis
  ○ Shortcomings in out‐of‐hours mental health care sees an over reliance on police officers managing community‐based mental health interventions. The Emergency Department is frequently accessed by police officers as a place of safety to support people in mental distress.
  ○ People in mental distress can experience their needs not being met by out‐of‐hour services.
- What the paper adds to existing knowledge?
  ○ Police and health care systems are perceived as disconnected and ill‐equipped to safeguard people who do not require inpatient care. Yet people in mental distress can feel unsupported in the community. Their mental distress brings them to the attention of the Police and the Emergency Department. Yet, there does not appear to be a smooth, dignified, cross‐ organisational response to their needs. This is particularly challenging for people who are intoxicated and need support out of hours.
- What are the implications for practice?
  ○ Experiences of people in mental distress during police and healthcare practitioner safeguarding vary. Access to services, diagnosis, sobriety, levels of aggression and professional attitudes are influential. These elements should be considered in the reimagining and planning of interagency mental health pathways to support people in mental distress who do not require inpatient care. Unscheduled care and community mental health nurses are well placed to influence the redesign of cross‐organisational mental health pathways.

## 1 Introduction

Internationally, there are increasing demands on police and emergency health services to respond to people in mental distress (PiMD) (Hudson et al. 2024; Livingston 2016; Sondhi and Williams 2018; Watson, Heyman, and Thomas 2021; Watson, Owen, et al. 2021), Yet there can be gaps in response linked to the complex nature of police demand, the scope of unscheduled mental health services, social exclusion and inequalities. This requires a strategic and tactical response, designed and delivered in partnership, at a local, regional and national level (HMICS 2023; Bath et al. 2021). To date, research and practice has frequently been focused on the police/PiMD/health intersect and sought to decriminalise people with severe mental disorders through diversion to psychiatric inpatient services (Ogloff et al. 2007; Harmon‐Darrow et al. 2023; Bennett et al. 2011; Hensen et al. 2016), mental health care in police custody settings (Hopkin et al. 2020; Cummins 2012) or models of police and health collaboration (Heyman and McGeough 2018; Heffernan et al. 2024; Marcus and Stergiopoulos 2022; Callender et al. 2021; Kane et al. 2017; Dawson and Hobson 2021).

Police officers use a range of responses when managing situations involving legislative powers to transport people to care. A literature review by Borschmann et al. (2010) examined the pathways of people detained under S136 of the English Mental Health Act (1983). Many people detained had been held previously under S136, suggesting that police officers play an ongoing role in mental health crises. Most studies reported a strong positive correlation between the police officers' beliefs about a person's mental state and corresponding psychiatric assessments, with the high rates of people detained and admitted when brought to hospital by police. This could suggest police officers can recognise accurately the signs and symptoms of serious mental illness. Furthermore, police officers could have a higher tolerance of unusual behaviours in the community, only bringing to hospital people who are seriously unwell.

In contrast, the Mental Welfare Commission (Scotland) (2018) reported in 2016 that 97% of people were not detained in a hospital when brought to a Place of Safety by police under their powers of detention of Section 297 of the Mental Health (Care and Treatment) (Scotland) Act (2003). Despite cautions over police recording practices potentially significantly underestimating the scale and severity of PiMD‐related demand (Langton et al. 2021), similar patterns are seen in England and Wales, with a six‐fold increase in police mental health concern referrals (Keown 2013). Nevertheless, there is a decline in the number of people admitted to hospital when brought to health services by police (House of Commons Home Affairs Committee 2015). This suggests a group of people who come to police attention for whom they are seriously concerned yet fall between the thresholds for inpatient psychiatric care and community‐based services, particularly during out‐of‐hours. It can be argued that current processes and professional partnerships are insufficient to meet the needs of the ‘missing middle’ (Thomas et al. 2022).

Intoxication can increase non‐compliance and violent behaviour, increase the severity of symptoms and lead to police detention (Chidgey et al. 2019). Although unusual, agitation, aggression and behavioural problems can see police officers rely on coercive interventions such as conducted electrical weapons or ‘Tasers’. These are more likely to be used on people experiencing mental distress than in cases of criminal arrest (Hallett et al. 2021). Yet, little is understood about the physical and psychological consequences of coercive interventions in this context (Hallett et al. 2021).

Adding to the complexity of care, prior contact with police, either as victim or perpetrator, is common for individuals in suicidal or self‐harm crisis (Chidgey et al. 2019). People who are intoxicated and distressed may be aggressive towards others, including police, bringing them to the attention of criminal justice services. Involuntary admission through the police is often traumatic, rooted in past police involvement in patients' lives (Iftikhar and Gorny 2022).

It is therefore important to understand the journeys of people where mental distress is not associated with a severe mental disorder or an offence. Previous literature reviews (Borschmann et al. 2010; Chidgey et al. 2019; Hallett et al. 2021) lack consideration of influences and the impact of organisational processes, professional perspectives, relationships and cultures on the experiences and safeguarding journey trajectory for PiMD.

The overall aim of this review was to determine current knowledge about the safeguarding journeys of PiMD supported by police and healthcare professionals (HCPs). In the context of this review, the term safeguarding is used to encompass these three perspectives, meaning the accessing of care and support by PiMD, and the experiences of police and HCPs in doing so.

The review question was:

What are the safeguarding experiences of people in mental distress, and what are the care experiences and processes of police and health practitioners in supporting PiMD needs?

## 2 Method

### 2.1 Integrative Review Process

This integrative review followed the rigorous four central steps identified by Whittemore and Knafl (2005): searching the literature, extracting, analysing, synthesising data and presenting the findings.

### 2.2 Data Searching and Extracting

Ten databases were initially searched from across health and social sciences in December 2018. This was conducted by the first author (IH). The review was updated in January 2024. The databases searched were the Cumulative Index to Nursing and Allied Health Literature (CINAHL), Web of Science, Medical Literature Analysis and Retrieval System Online (MEDLINE), The Cochrane Collaboration, Applied Social Sciences Index and Abstracts, Database of Reviews of Effects (DARE), PsycInfo, EMBASE, Psychology and Behavioural Sciences Collection (PSBC), National Criminal Justice Reference Service and Google Scholar. Hand searching included studies appearing in books, published and unpublished works, conference proceedings, related citations and reference lists of relevant papers. Search alerts were also set up for each of the databases to ensure up to date literature was included.

**FIGURE 1:**
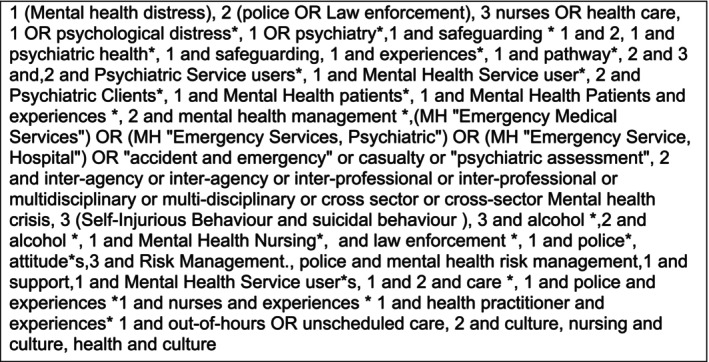
Literature review key search terms.

The search terms (Figure 1) were refined and adapted following the initial search. In the initial search, inclusion dates were 1st January 2002–December 2018. The review was updated in January 2024 using the same original search terms. The dates were chosen to include contemporary key legislative and policy changes in the care of PiMD such as the introduction of the Adult Support and Protection (Scotland) Act (2007).

The review question seeks to address gaps in knowledge in experiences and processes drawing on a range of primary qualitative, quantitative and mixed methods sources. Included in this integrative review are evaluations of inter‐agency experiences between police, emergency health services and PiMD, original qualitative and quantitative papers written in the English language. Excluded were papers specifically reporting on children (under the age of 16), hospital inpatients, other forensic settings, such as prisons, police custody settings (other than issues relating to processes of safeguarding procedures) and papers with an identified focus on people with severe and enduring mental illness.

### 2.3 Management and Selecting of Key Literature

**FIGURE 2:**
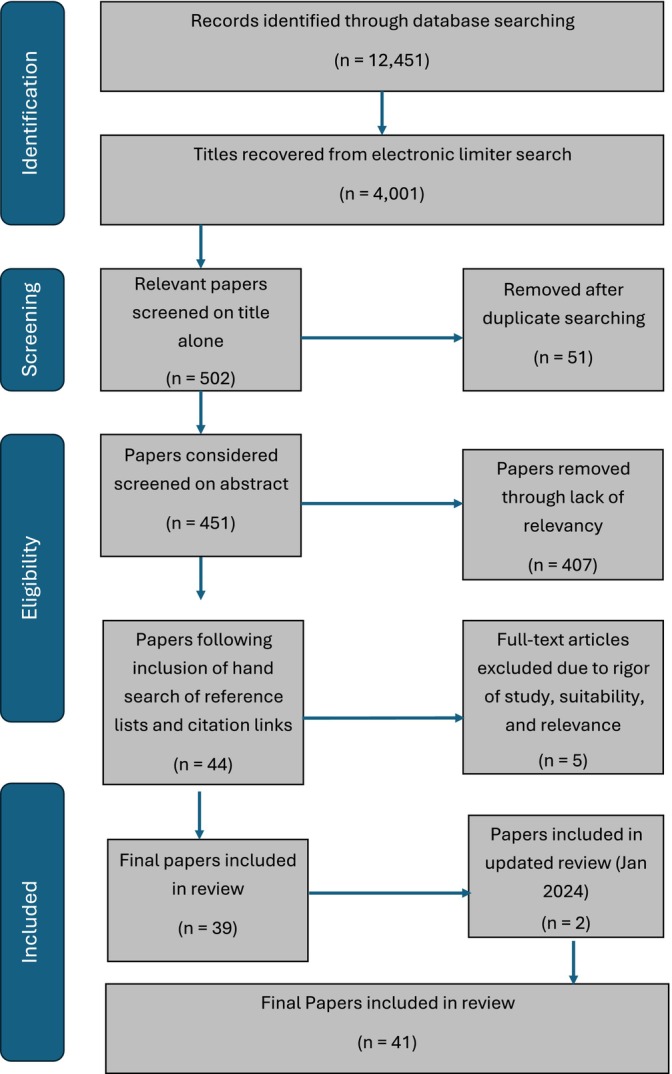
PRISMA flow diagram.

Key search terms were applied (Table 2). Citations, abstracts and full text articles were collated. This process is presented in a PRISMA flow diagram (Adapted from Page et al. 2020) in Figure 2.

### 2.4 Quality Appraisal

The Critical Appraisal Skills Programme (CASP) (Critical Appraisal Skills Programme 2018) supported a systematic quality appraisal of qualitative studies. Quantitative and mixed‐method studies were assessed using a tool informed by Crombie (1996) in which the quality of each paper was scored according to specific criteria. The maximum score (indicating high quality) was 16, with the lowest possible score being zero. Each study was subsequently rated as low (0–5 points), moderate (6–11 points), or high (12–16 points). The CASP tool of 10 questions also rated papers as high/medium/low. Scores for each paper are indicated in Appendix S1: Literature review data extraction summary table.

### 2.5 Thematic Analysis

Each paper was read several times with key findings extracted and recorded using a spreadsheet (Miles and Huberman 1994). This approach supported the organisation of literature to facilitate data synthesis. Initially, the synthesised findings reflected the original findings of the included studies. The findings of each study were combined into a whole via a listing of themes, which reflected PiMD, presentations, policy, police and health professionals' experiences. The initial synthesis did not directly address the review question concerning what is known of peoples' experiences, care and safeguarding processes between the services.

The next step involved identifying and coding findings from each study to construct descriptive themes. Initially, 13 descriptive themes were identified: risk tolerance; service demands; complexity; the multiplicity of the process; protecting communities; protecting the individual; inter‐agency working; siloed working; relationships; professional attitude; professional cultures; risk and trust; and professional disparities. The final stage involved returning to the review question with the descriptive themes to allow the emergence of abstract or analytical themes. This synthesis was developed through extensive review with the authors until the three key themes were abstracted and captured.

### 2.6 Findings

Of the 41 papers included, 16 papers were from Australia. Five were from Canada, six from the U.S.A., five from England, two from Scotland, three from Ireland, one from the Netherlands, one from New Zealand, one from Belgium, plus two international studies.

Of these, 20 were qualitative papers, 19 were quantitative papers and 3 mixed‐methods studies. The majority of the qualitative studies used semi‐structured interviews. Two papers used focus groups, and one was an observational study. Eleven qualitative studies were concerned with HCP experiences or attitudes of supporting people who self‐harm or of experiences of people referred by police. Four papers were concerned with PiMD experiences of Emergency Department (E.D.) visits, and three reported experiences of being supported by police officers. Of the quantitative papers, 11 used a cross‐sectional survey design and one Delphi study. Twelve studies included retrospective review or audit of police or E.D. records. The majority of retrospective records reviews were concerned with characteristics of PiMD attending the E.D. or coming to police attention. Co‐morbid distress and intoxication were factors in five papers. Potentially due to the nature of PiMD safeguarding, no randomised controlled trials were identified.

Synthesis of papers and quality scores ordered in three overarching themes by the first author (IH) are found in data extraction table A (Appendix S1).

Three overarching themes emerged:

Safeguarding and care experiences of people in mental distress (11 papers): Intoxication, self‐harm and aggression (11 papers): Professional perspectives and responses to PiMD (22 papers).

Four papers (Doyle et al. 2007; Watson et al. 2008; Chapman and Martin 2014; O'Keeffe et al. 2021) were assigned across two themes.

### 2.7 Safeguarding and Care Experiences of People in Mental Distress

PiMD experiences of police or emergency services care were reported in 10 studies. Two subthemes emerged. First, ‘Using the E.D. for mental health care in seven studies’, and second ‘Experiences of the police/and or emergency health services’ in three studies. The literature highlights the crisis nature, experiences, quality and accessibility of care for PiMD in the E.D. is variable.

Experience of using the E.D. for mental health care is reported in seven papers (Bruffaerts et al. 2006; Digel Vandyk et al. 2018; Brunero et al. 2007; Spence et al. 2008; Kuehl et al. 2012; Joubert et al. 2012; O'Keeffe et al. 2021). In terms of help‐seeking, the E.D. is used by people with a wide range of mental health needs and an important entry point to mental health care due to accessibility. Bruffaerts et al. (2008), found the E.D. had become a critical point of contact for people with common mental health problems such as mood and anxiety disorder. Six in ten people used a psychiatric emergency room (PER) within a general E.D. for the first time as part of their mental health help‐seeking journey. These data suggest there may be limitations to timely community mental health care, with the E.D. as the primary support option.

Psychiatric diagnosis was influential in recurrent E.D. attendance for a small subset of people. Digel Vandyk et al. (2018) highlight utilisation patterns according to psychiatric diagnosis by people with 12 or more visits to the E.D. Participants reported they felt compelled to attend, and every visit was necessary, yet they felt dismissed by E.D. staff. Participants with primary personality disorders reported they hated visiting the E.D. but felt they had nowhere else to go to keep themselves safe. In contrast, people with a psychotic disorder diagnosis viewed the E.D. as a safe place and felt they were treated by staff as needing support. This suggests diagnosis and E.D. staff perception of need can influence attendance, experiences and outcomes for PiMD. Similarly, Spence et al. (2008) found men who self‐harmed and used substances (*n* = 25) reported frequent use of the E.D. due to a lack of community‐based services. However, they felt their needs were beyond the purview of the E.D. This highlights the system in which people seek safety can fail them (Holm and Severinsson 2011). Potentially, this can contribute to cycles of distress and repetitive support seeking through emergency care.

Similarly, O'Keeffe et al. (2021) report PiMD felt they received cyclical crisis support. For some, this meant they were signposted back and forth between the Emergency Department and crisis team, with nothing in place in the community to avoid reaching crisis point and returning repeatedly to the E.D. Yet this was underscored by feelings of shame and guilt for seeking support in the E.D. when at risk of self‐harm.

PiMD who recurrently require E.D. mental health care are more likely to be transported by police. In Australia, Brunero et al. (2007) found more police referrals among PiMD who attended between two and three times in 12 months. Recurrent police referrals may be explained by some PiMD coming to police attention for other reasons. There is strong evidence of illicit substance use co‐morbidity, domestic violence and homelessness linked to self‐harm and police attendance (Van Dijk et al. 2019; Kothari and Rhodes 2006; Hodges et al. 2006; Saddichha et al. 2014). Such complex problems can find some people unable to change their circumstances, perpetuating frequent police contact and subsequent discharge back to the community.

Focusing on the individual may not provide a full picture of recurring mental distress presentations to the E.D. Four papers (Spence et al. 2008; Kuehl et al. 2012; Joubert et al. 2012; O'Keeffe et al. 2021) point to systemic problems such as a lack of community‐based services forcing people to repeatedly use emergency services as a last resort to keep themselves safe.

Re‐presentations can be rooted in systems failures (Kuehl et al. 2012). A retrospective records review from New Zealand identified a small group of people rapidly re‐presented to the E.D. within 24 h following intentional self‐harm. Of the 73 re‐presentations, more than half (55%) occurred within 24 h of the index presentation. The authors suggest limited mental health assessment and inadequate discharge as reasons for representation. Thus, there appear issues with systems within the E.D. which can contribute to repeat attendance.

By way of contrast, the E.D. can establish connection to community mental health support for people following the first onset of emotional problems. In Australia, Joubert et al. (2012) identified linkage back to the community with most patients (78%) receiving care in the E.D. Whilst some PiMD feel they are not a priority and ‘batted back’ home (Barratt et al. 2016), Joubert et al. (2012) identify a need to ‘keep’ people to allow comprehensive assessment and care planning before discharge. Given the high number of people previously identified who present without previous mental health care, this study appears to connect effectively more people to care and reduce transitory journeys through the E.D.

Yet, there is recognition that emergency services are not fully equipped to deal with the complexity of PiMD needs. How people experienced police and HCP collaboration was discussed in three international papers (Watson et al. 2008; Wise‐Harris et al. 2017; Clarke et al. 2007). Few studies have examined PiMD experiences of the police intersecting out with the collaborative models.

Police officer role and their approach to keeping people safe can impact on individuals' experiences of safeguarding. Watson et al. (2008), in a qualitative study in the USA, explored the experiences of PiMD (*n* = 20) in 67 encounters with police. Although participants encountered police in a variety of ways, two main themes emerged. First, PiMD can feel vulnerable and fearful of police, and second, the way police treated them mattered. Adverse experiences were abuse from police officers and the absence of a voice. The authors point to police being rushed and using force to manage incidents. In contrast, positive experiences were being treated with kindness, dignity and being heard.

PiMD can experience a similar lack of kindness in health services. Wise‐Harris et al. (2017), a mixed‐methods Canadian study, reports that people with mental health and/or substance use challenges experience negative encounters due to a lack of ‘fit’. PiMD in generalist E.D.s can experience stigma, discrimination and unsympathetic care. The authors call for appropriate training and support for HCPs to address complex physical and mental health needs.

In contrast, in another Canadian qualitative study held with PiMD and their families, Clarke et al. (2007) found participants universally wished to be seen in a generalist E.D. and not at a separate psychiatric service. This was due to concerns of psychiatric facility stigma and an inability to deal with physical care. These studies underscore stigma for PiMD is experienced across a range of contexts. This theme suggests diagnosis, perceptions and complexity of individuals needs and being treated with humanity are influencing factors in the experiences and outcomes for PiMD coming to the attention of police and/or attending the E.D.

### 2.8 Intoxication, Self‐Harm and Aggressive Behaviours

A second key theme is associated with the experiences of police and HCPs in the management of PiMD, who were intoxicated or aggressive and was reported in 11 papers; (Borges et al. 2006; Larkin et al. 2017; Griffin et al. 2017; NHS Quality Improvement Scotland 2008; Downes et al. 2009; Zisman and O'Brien 2015; Maharaj et al. 2013, 2011; Morphet et al. 2014; Doyle et al. 2007; Lord and Bjerregaard 2014). Evidence suggests co‐occurring intoxication and aggression are commonplace in emergency responses to PiMD and can impact care, as well as police and E.D. resources (Xuan et al. 2016; Johansen et al. 2010; Bagge et al. 2017).

There is a call for a tailored approach when PiMD are intoxicated to minimise the risk of further non‐fatal or fatal self‐harm. A World Health Organisation quantitative study of 10 E.D.s (*n* = 4320) by Borges et al. (2006) found that the risk of self‐injury increased tenfold after six units of alcohol. These findings support other studies in finding alcohol as an independent indicator for suicide and self‐harm (Larkin et al. 2017; Ames 2017; Griffin et al. 2017). Similarly, in Scotland, an NHS Quality Improvement Scotland (2008) audit into harmful drinking found that more than half of those with self‐harm injuries had consumed alcohol prior to attending the E.D. Around 27% of men and 19% of women cited alcohol as a trigger for self‐harming, supporting evidence intoxication as a key co‐occurring factor in the management of PiMD presenting through emergency services.

The impact of intoxication on cognition and behaviours, such as violence and aggression, can be the catalyst to bring people to the attention of police and health services. Understanding of community‐based aggressive behaviours associated with self‐harm and intoxication is limited in the policing literature despite how often this occurs. Behavioural disturbance is common in the E.D. and has received slightly more attention. Downes et al. (2009) Australian retrospective review of acute behavioural emergencies (*n* = 143) requiring a specialist hospital violence response team suggests aggression in people presenting with self‐harm (38%), alcohol and illicit drug intoxication (33%) and psychiatric illness and drug withdrawal (29%). Unknown from this study are any identified reasons behind the aggression, for example long wait times (Morphet et al. 2014). In summary, Downes et al. (2009) suggest co‐occurring intoxication and violence bring an additional challenging behaviour for emergency services when managing the care of some PiMD.

Police referrals of PiMD who are intoxicated to psychiatric services is commonplace. Zisman and O'Brien (2015) explored the relationship between alcohol and other substance use, and the process and outcomes of detentions under S136 of the English Mental Health Act. Over a 6‐month period, 245 individuals were assessed. Threatening self‐harm (*n* = 100, 44.8%) was the most common reason for an assessment. Those transported by police had high rates of intoxication with alcohol or other substances (69.5%, *n* = 66) and longer assessment times. Given the previously reported elevated risk of serious self‐harm associated with drunkenness, it is concerning that those who are intoxicated are significantly more likely to be discharged home than admitted to hospital, indicating they did not need emergency psychiatric services. However, of those discharged, the majority (61.5%, *n* = 83) were intoxicated at the time. A limitation of this retrospective study is that data were drawn from electronic notes. This can bring into question the accuracy of recording and reporting bias. It does not explain how people who were intoxicated were managed whilst awaiting assessment, or how they returned home, for example, by police escort.

In contrast, a comparative Australian retrospective audit (Maharaj et al. 2011) found those referred by police were more likely to be intoxicated, yet more likely to be admitted to the psychiatric unit. People referred by police had significantly higher rates of mental distress and aggression because of psychoactive substances, compared to those not seen by police. Potentially, PiMD who are intoxicated or have aggressive or unusual behaviours because of substances are more likely to come to the attention of police. Potentially, also, the difference in outcomes for PiMD referred by police reflects different agreements between police and health services as to whose responsibility it is to manage and safeguard people who are intoxicated.

Compared to the previous study (Maharaj et al. 2011), PiMD referred by police were more likely to be discharged after a few days than people referred by other sources, suggesting that their mental health needs may be linked to co‐occurring substance use, rather than a mental health problem alone (Zisman and O'Brien 2015). It may also signal there is recognition that to keep people safe when intoxicated, they may benefit from inpatient care.

As well as difficulties with challenging behaviour due to intoxication, intoxication can compromise clinical assessment of mental well‐being and risk. Co‐occurring intoxication and self‐harm can delay decision‐making and challenge supervision of people in clinical environments (Yost 2002). In part, this is due to lengthy wait times awaiting PiMD sobriety and availability of a psychiatrist to conduct a mental health assessment. A Delphi study by Morphet et al. (2014) suggests the combination of long waiting times for assessment, drugs and alcohol are highlighted as key contributors to violence in the E. D. Thus, there appears to be a relationship between psychiatric assessment procedures, intoxicated behaviours and lengthy wait times which can impact on PiMD experience in the E.D.

Another reason PiMD who are intoxicated may be escorted by police in the E.D. is that there can be a risk PiMD leave before they are assessed (Griffin et al. 2017). This behaviour may be partly in response to delayed assessment whilst awaiting sobriety. Given the increased risk of serious harm or suicide associated with mental distress and intoxication (Olson et al. 2013; Spence et al. 2008), these findings are important to suicide prevention initiatives.

In an analysis of data on self‐harm presentations to hospital E.D.s in Ireland and Northern Ireland, Griffin et al. (2017) found 43% of people presenting with self‐harm were intoxicated. This group were more likely to leave the E.D. without being seen by a clinician. Nurses felt ill‐equipped to care for the complex needs of this population; such cases being more likely to occur out of hours when there were fewer resources to manage such behaviours.

Similar findings were reported in an Irish qualitative study of nurses (*n* = 42) experiences of caring for PiMD in the E.D. (Doyle et al. 2007). Participants reported a key challenge working with this group was preventing the patient absconding and acting on further self‐harm. Nurses reported becoming hyper‐vigilant and draining resources in an already busy clinical area. Nurses in this study sometimes felt uneasy and stressed when caring for those who were aggressive, not equipped to manage PiMD, and out with their role.

Two papers identify aggressive behaviours of PiMD referred to health services by police (Lord and Bjerregaard 2014; Maharaj et al. 2013). In the United States, Lord and Bjerregaard (2014), examination of 3635 cases in police and health files in the E.D., revealed police referrals to psychiatric emergency services are different from those referred from other sources and were significantly more likely to be volatile. Similarly, in Australia, Maharaj et al. (2013), qualitative study points to prominent levels of aggression in PiMD referred by police officers to psychiatric services. People were stereotyped by nurses as ‘the worst’ patients and easily distinguishable by their aggressive behaviours. Nurses dichotomised people referred by the police as ‘deserving’ and ‘undeserving’ of care. People with ‘genuine mental illnesses’ were believed to be deserving. The salient features of the ‘undeserving’ people were that they were drug and alcohol affected, demonstrating suicidal and threatening behaviour.

Professional perspectives and responses to PiMD experiences of safeguarding PiMD was reported in 22 papers. No studies focused on both HCP and police perspectives; therefore, this theme is presented in two sub‐themes being the HCP and police perspectives. Furthermore, no studies have considered these views together. This theme will conclude with a discussion of findings associated with professional relationships, organisational processes and professional cultures.

Health care professional perspectives and experiences of safeguarding is discussed in 12 papers (McAllister et al. 2002; Conlon and O'Tuathail 2012; Chapman and Martin 2014; Doyle et al. 2007; Commons‐Treloar and Lewis 2008; Betz et al. 2013; O'Keeffe et al. 2021; McCann et al. 2006; Friedman et al. 2006; Thompson et al. 2008; Summers and Happell 2003).

The literature suggests a relationship exists between HCP attitudes, beliefs and behaviours about self‐harm and their interactions with PiMD. In this section, HCP attitudes and experiences of supporting PiMD are reported in 10 papers. Studies measured and explored factors influencing HCP attitudes such as professional experience and related concepts such as perceptions of the ‘genuineness’ of the individual's needs.

PiMD, who do not receive positive, empathetic and caring attitudes, are less likely to remain in the E.D. for treatment (McAllister et al. 2002). Nurses' attitudes to people who self‐harm appear shaped by judgements made on the act of self‐harm itself. Through a quantitative questionnaire, Conlon and O'Tuathail (2012) sought to measure Irish nurses' (*n* = 87) attitudes towards self‐harm using the Self‐Harm Antipathy Scale. The authors contend self‐harm is frequently judged by nurses as morally wrong. This implies critical judgements are made about help‐seekers. This is possibly due to whether nurses distinguish behaviours being individual choice or a response to mental illness. In other words, if nurses felt these behaviours could be alleviated by a clinical intervention, then they may act more positively towards the individual.

Similarly, judgements are made by emergency medicine clinicians on the ‘genuineness’ of the individual in the frequency of attendance and the type of harm. Chapman and Martin (2014) reported in an Australian qualitative study that staff experienced PiMD to be manipulative. Some clinicians clearly differentiated between those wishing to die by suicide compared to those labelled as ‘attention‐seeking’. Although some report feeling empathetic towards people who deliberately self‐poisoned and felt they treated all patients the same, many participants expressed frustration with this population. These findings mirror the experience of PiMD identified earlier where they felt they were often not taken seriously. Therefore, it could be unhelpful and potentially dangerous if E.D. clinicians hold a belief that a PiMD is ‘attention‐seeking’.

Several authors (Doyle et al. 2007; Commons‐Treloar and Lewis 2008) cite frequent presentations of the same person with increased pessimism, loss of empathy and consequently, the development of negative attitudes in E.D. clinicians. Exposure to repeat presentations has been reported as reinforcing beliefs and doubt about the likelihood of PIMD going on to die by suicide. Some clinicians reflect scepticism about the preventability of suicide, shifting the focus from the individuals' behaviours to the confidence and skills of clinicians to intervene with PiMD (Betz et al. 2013). In a multi‐site survey of 8 E.D.s in the U.S.A., Betz et al. (2013) found few physicians and nurses (*n* = 631) believed that suicidal patient treatment was a top priority. Yet, participants reported frustrations over gaps in their risk assessment skills and provision of referral resources to prevent repeat presentations. These findings are concerning given the literature presented earlier in this review identifies people may not have met their needs and can remain at risk of repeat self‐harm or suicide after attending the E.D.

Similarly, O'Keeffe et al. (2021) report E.D. practitioners can feel powerless to support people who self‐harm with recognition of the complexity and long‐term nature of individuals' needs. In this qualitative study, practitioners felt powerless to stop self‐harming. Such powerlessness is linked to practitioners' negative attitudes when people re‐attend, burnout and becoming ‘hardened’ or ‘cold’ towards people. This in turn intensified feelings of worthlessness of those seeking support.

In contrast, positive attitudes of community mental health nurses and E.D. nurses are influenced by nurses' experience and education associated with self‐harm (McCann et al. 2006; Friedman et al. 2006; Thompson et al. 2008). These studies report that older and more experienced nurses demonstrate more positive attitudes compared to younger colleagues. This may be linked to confidence developed through experience in the assessment and management of PiMD. These findings indicate that whilst these nurses did express frustrations over repeat presentations, they held sympathetic attitudes towards PiMD and did not discriminate against them in their triage and care decisions.

It is also proposed that increasing therapeutic and interpersonal communication in, and directly after, presenting to the E.D. could be beneficial for PiMD (Summers and Happell 2003). This suggests staff knowledge, experience and skills can influence PiMD experiences of care and potentially increase engagement with services.

#### 2.8.1 Police Officers' Perspectives and Experiences of Safeguarding

Fifteen papers focused on police officers' attitudes and experiences in supporting PiMD. These tended to differ from those in the HCP studies in the previous section, in that for the most part, the emphasis of these papers was on frustration over practical tasks and access to health services, rather than attitudes towards PiMD per se.

Eight papers reported police officers' difficulties transferring care of PiMD to health services (Godfredson et al. 2011; Watson, Heyman, and Thomas 2021; Watson, Owen, et al. 2021; McLean and Marshall 2010; Fry et al. 2002; Al‐Khafaji et al. 2014; Martin and Thomas 2015; Watson, Heyman, and Thomas 2021; Watson, Owen, et al. 2021; Lamb and Tarpey 2019). Godfredson et al. (2011) conducted a qualitative survey of 3534 Australian police officers to explore the ‘approach styles’ of police when responding to PiMD. Officers expressed frustration at having to ‘babysit mentally ill people’ in hospital waiting rooms, whereas others found the mental health system to have a ‘revolving door policy’. This suggests people can be released from health services only to return to the attention of police officers.

Police officers report help from HCPs is unavailable in a timely, or even in an urgent manner (Fry et al. 2002; Lamb and Tarpey 2019). Similar challenges of lengthy wait times and difficulties in discharging care to health services are echoed in a Scottish study (McLean and Marshall 2010). Based in a large urban area of Scotland, this qualitative study of semi‐structured interviews with police officers (*n* = 9) reported they felt there can be an inappropriate burden placed upon the police service to support PiMD. This impression was reinforced by a belief health services failed some vulnerable people. Police found themselves supervising people on ‘suicide watch’ within custody suites, rather than in health services, drawing them away from ‘real police work’.

Police officer engagement with health services appears more challenging when transferring care of some PiMD and after 5 pm (Martin and Thomas 2015). Martin and Thomas (2015), in an Australian qualitative study, sought to examine police encounters with people with mental health needs through semi‐structured interviews (*n* = 25). A key finding from this study was that officers specifically identified difficulties in engaging HCP support for people with a diagnosis of personality disorder (PD). Like Chapman and Martin (2014), reported in the previous section, participants in Martin and Thomas's study reported HCPs labelled this population as ‘attention‐seeking’, thus leaving police officers with ethical dilemmas and frustrations in keeping people safe when there was no health care support. Similarly, two qualitative studies explored police officer experiences in responding to PiMD. First, in England (Lamb and Tarpey 2019) and second in the USA (Wood et al. 2021; Watson, Heyman, and Thomas 2021; Watson, Owen, et al. 2021) officers expressed concern about gaps in health and social care services finding the unmet needs of some people, placing responsibility on police services to provide care. These studies draw attention to a gap in care for people in mental distress with a diagnosis of PD, making connections between HCPs beliefs in their abilities to intervene and police officers' abilities to transfer care.

In most countries, police can use their powers under mental health legislation to facilitate access to mental health care. A retrospective medical review by Al‐Khafaji et al. (2014) sought to understand the characteristics of patients brought by police to health services under mental health legislation in Australia. This legislation attempts to balance public safety and timely access to mental health care for people who police believe are mentally unwell. Yet police detention and involuntary transport can come at a cost to personal freedom and physical and psychological risk. Al‐Khafaji et al. report 61% of people did not require restraint, sedation or hospital admission and 67% were discharged home, suggesting they did not require this level of detention. In 1.6% of cases, there was no evidence in the police documents of threat/risk to self or others. These cases would appear to fall outside the provisions of mental health legislation (Al‐Khafaji et al. 2014).

There is evidence also of other emergency services ‘leaning in’ on police officers to use their powers of detention to facilitate access to mental health care for some PiMD. In England, Rees et al. (2016) investigated paramedic responses to PiMD. Paramedics reported situations where there were tensions between legality and good practice when caring for people who refused transportation to a hospital. Calls for support by police were reported as standard practice for paramedics as a means of using police powers to detain a person and transport them against their will. This practice reflects ways in which services work around gaps in systems yet underlines that this may come at the expense of the dignity of PiMD and may breach ethical and legal principles.

International literature reflecting a failure on the part of HCPs to hospitalise PiMD caused considerable angst among police (Fry et al. 2002; Schulenberg 2016; Godfredson et al. 2011). Schulenberg (2016), in a Canadian mixed‐methods study, found officers wished to be part of a solution to keep people safe and advocate for diversion from the criminal justice system where possible. However, in too many circumstances, police officers were faced with arrest decisions for public order behaviour when unable to discharge care to HCPs, thus laying criminal charges for minor offences due to limits on their decision‐making autonomy (Schulenberg 2016).

Cotton (2004) concurs, finding Canadian police officers face complex situations, and their decision‐making operates in a ‘grey zone’. Cotton (2004) suggests police officers are in an untenable position. There is a social expectation to ‘do something’, whereas at the same time having no clear reason to arrest and knowing full well a visit to the E.D. is unlikely to lead to admission or treatment, unless the individual is acutely homicidal or actively suicidal.

Challenges in balancing law enforcement and social welfare roles, when called on to safeguard PiMD, were also reported in three studies. For the most part, there is evidence police officers feel compassion and understanding of PiMD with feelings of having made a positive impact on people (McLean and Marshall 2010; Lamb and Tarpey 2019). Godfredson et al. (2011) found Australian police officers expressed empathy for PiMD, a desire to protect them and an enthusiasm by police officers for mental health training to improve care. Yet, applying mental health skills in police practice was found to be challenging. This was because a culture of doing ‘real police work’, such as crime‐fighting, can be strong, and health services were being under‐resourced to support police referrals (Godfredson et al. 2011; Watson, Heyman, and Thomas 2021; Watson, Owen, et al. 2021). Thus, despite a willingness to improve police responses to PiMD, in practice inter‐agency systems and police culture can impact upon PiMD support.

Positive police officer experiences in the management of PiMD, on the other hand, are associated with connecting PiMD to health services or managing less urgent distress without transfer to mental healthcare or criminalisation. Van Den Brink et al. (2012), in a Dutch study of police records, suggests police are experienced conduits to mental health services. Half of PiMD coming to their attention were not previously engaged in psychiatric services, yet police officers were responsible for connecting a substantial portion of individuals (21%) with mental health services. Half of all encounters were dealt with by police alone. This study did not illuminate how PiMD were managed, or the outcomes of intervention, yet Van den Brink suggests officers in the Netherlands have a greater level of agency and discretion associated with their approach to PiMD. They appear confident in their ability to deal with mental health issues beyond arrest or referral to health services. Van Den Brink's study underscores that this does occur in some jurisdictions; however, officers' discretion in mental health care was not identified in other papers reviewed.

Viewing professional experiences of safeguarding PiMD together, there is a suggestion of significant tensions in the transfer of care of PiMD from police to emergency health services. This appears due to gaps in emergency safeguarding environments and interagency processes for people who do not require inpatient care, yet whose safety requires police intervention. Such gaps, tensions and cyclical processes reinforce negative professional perspectives of some PiMD and interprofessional distrust.

## 3 Discussion

Taken together, the findings of this integrative review illuminate the experiences of PiMD during safeguarding that are varied and multi‐faceted in nature. Although there is evidence of positive experiences where people are treated with kindness and compassion, a substantial number of the reviewed articles report negative experiences. These appear linked to tensions between the crisis nature of self‐harm and intoxication bringing people to the attention of police, emergency HCPs, professional responses to their needs, and the intersection of emergency police and health systems in which support is provided.

When comparing PiMD experiences, being heard, believed and treated with dignity are crucial factors in how PiMD experience safeguarding. These factors can be influenced negatively due to police and HCP responses to PiMD diagnosis, beliefs about self‐harm, the co‐existence of physical and mental health problems, and frequency of E.D. attendance. The review highlighted that experiences are particularly challenging for people with a diagnosis of personality disorder, PiMD who are intoxicated or aggressive. Police/health systems where safeguarding is poorly organised can contribute to poor care experiences. This draws into sharp focus the potential for diagnostic overshadowing and compromised care. Understanding PiMD needs during interprofessional safeguarding could go some way to preventing frequency of occurrence and negative outcomes for PiMD and services.

A key thread running throughout the papers was that police officers occupy an essential position in safeguarding PiMD. This is underscored in the focus of the studies in the integrative review with 15 of the 41 studies focused on the police experience. Police officers can be challenged in balancing their law enforcement and welfare roles. In part, this can be because of difficulties in being able to discharge the care of some PiMD to health services. Adding to this, police processes, such as legislative powers and restraint used to manage challenging behaviours and access to mental health support, can be experienced as frightening and coercive, potentially increasing agitation and aggression.

Unscheduled care and community mental health nurses have a vital role to play in recognising the impact of systemic gaps on the cyclical experiences of PiMD brought to emergency services by police. This in turn could further assist in reducing stigma, increasing safety, hope and dignity for this ‘missing middle’ inadequately recognised population.

The disparate professional perspectives of PiMD needs, professional relationships, organisational processes and professional cultures suggest there is also a significant role for interprofessional education in the preparation of police and healthcare professionals. Given the safeguarding experiences illuminated in this review, it is crucial that such interprofessional education is co‐produced with people who have experienced emergency mental health support.

Police officers and HCPs experience a high level of frustration in supporting PiMD. HCP frustrations appear aligned to PiMD behaviours, rather than those of health and police systems in managing PiMD. In contrast, police officers' frustration appears more focused on gaps in resources. Although police officers may wish not to criminalise PiMD, the inter‐agency police/health system can impede transfer between services, thus leaving few options open to police officers. There is a relationship between professional attitudes to PiMD, clinical knowledge, competing roles and available police and HCP resources. These appear to see systems work in opposition, highlighting failings in inter‐agency practices and systems to support some PiMD, thus supporting calls for targeted mental health models of care to better support people brought in by police to an ED (Wardrop et al. 2023).

## 4 Limitations

The searches in this review returned a high number of quantitative papers (*n* = 19) and may not fully reflect the focus on human experiences within the research question. Papers included empirical studies from 10 countries. These results should be interpreted with caution, given these were English language studies alone, and may not reflect the experiences from other populations and cultures. Data from 11 papers were drawn retrospectively from police or health records and could be subject to recording bias, thus limiting the quality of these studies. The screening of titles, abstracts and quality of the literature was conducted by the first author (I.H.) which may have resulted in bias. All authors (I.H., C.K., A.W., A.G.) collaborated on the remaining stages of the review.

Within included studies, there may be significantly different contextual issues, such as safeguarding policies, mental health legislation and cultural differences, making comparisons difficult and not easily transferable to other contexts. For example, many studies were conducted in the U.S.A. and Australia where there is limited comparison to access government‐sponsored universal healthcare systems such as that of the UK, and much higher rates of mental health‐related exchanges with police involving firearms. These could potentially influence PiMD and professional experiences.

## 5 Conclusion

This review stresses gaps in emergency health and police processes to work in tandem to effectively support the needs of some PiMD. Specifically, these gaps impact on PiMD where inpatient care is not required, yet their well‐being and safety are sufficiently concerning for police to seek health care support. The recurring and crisis nature of mental distress, coupled with a lack of out‐of‐hours community‐based care, appears to find PiMD reliant on police and emergency services to support their needs (Watson et al. 2008; Wise‐Harris et al. 2017; Clarke et al. 2007). Yet, both police and the emergency health care systems appear ill‐equipped to support the needs of this ‘missing middle’ population. This can find people returning to their communities and disconnected from primary health services.

There is an urgent need to re‐design, develop and test alternative safe spaces to support the people who come to the attention of police and other emergency services. Redesign could prevent recurrent presentations of those who may be vulnerable because of cross‐organisational system gaps within the current medicalised model of emergency care. Mental health nurses should have a voice in re‐imagining such care and could go some way to supporting a more dignified and effective unscheduled care service.

Provision of safe, dignified and compassionate care sits at the heart of interagency safeguarding practice. Yet, there remains a poor understanding of how systematic gaps relate and shape key stakeholder experiences during mental distress. Future research and interprofessional education may be wise to focus on understanding the nuance and trajectory of the safeguarding journey to build a comprehensive picture of barriers and facilitators towards keeping PiMD safe and consider allocation of police and health resources. Better understanding of the interconnectedness between systems gaps and human inputs within these gaps can help illuminate factors which can support or act as stressors to mental distress during out‐of‐hours safeguarding journeys.

## Conflicts of Interest

The authors declare no conflicts of interest.

## Supporting information


**Appendix S1:** jpm70014‐sup‐0001‐AppendixS1.docx.

## Data Availability

The data that support the findings of this study are openly available in OPEN AIR @RGU at https://rgu‐repository.worktribe.com/output/1357998/people‐in‐mental‐distress‐police‐and‐out‐of‐hours‐health‐services‐a‐qualitative‐exploratory‐case‐study‐of‐experiences‐and‐the‐intersect‐of‐safeguarding‐services.
