# Supplementary material for: Safeguarding Experiences of People in Mental Distress, Police and Healthcare Practitioners: An Integrative Review
Source: J Psychiatr Ment Health Nurs. 2025 Aug 14;32(5):1262–74. doi: 10.1111/jpm.70014 (PMC12418150; doi:10.1111/jpm.70014)
Supplement: Supplementary file 1 — Appendix S1: jpm70014‐sup‐0001‐AppendixS1.docx. [file JPM-32-1262-s001.docx]

| Theme 1. Safeguarding and care experiences of people in mental distress | | | | | | | |
| --- | --- | --- | --- | --- | --- | --- | --- |
| Authors | Country | Objectives | Research Type | Research Method | Sample Size | Results | Key findings |
| Bruffaerts et al. (2006) | Belgium | To examine patient and system characteristics of first-time ("incident") vs. recurrent ("recurrent") use of a psychiatric emergency room (PER) | Mixed methods    Quality score 12 | A semi-structured interview  based on the Minimal Psychiatric Data form, a standardized and  validated psychiatric patient registration form  used to gather information on patients’ demographic and clinical  characteristics and mental health service use | N= 3,719 | About 64% (n=2,368) were  incident and 36% (n=1,351) were recurrent users. The  PER was the first treatment setting ever for 50% of the  incident users. Incident users were most likely over  69 years or referred by a health care professional. They were less likely to have a personality disorder or to have used inpatient or outpatient services in the past. About 44% were  admitted, 38% referred for outpatient treatment, 9%  referred to the outpatient crisis-intervention program,  and 9% refused any follow-up. | The Psychiatric emergency room was a first treatment setting ever for 1 in 3 patients. Incident and recurrent users differed in sociodemographic characteristics, pathways to care, service use,  and the presence of a personality disorder. They did not differ in axis 1 disorders, comorbid mental disorders, or pathways after care. |
| Digel Vandyk (2018) | U.S.A. | Explore the experiences of persons who frequently present to the ED for mental health-related reasons. | Qualitative  Quality score High | Interpretive Description   A using semi-structured interview and  a simple sociodemographic survey | N= 10 | Frequent presenters to the ED for mental health-related  reasons feel compelled to come to hospital. For them,  every visit is necessary, and dismissal of their needs by  ED staff is interpreted as disrespect and prejudice. A lack of adequate discharge planning upon release from the ED appears to perpetuate further ED use, especially when safe transportation home is not available. | The participants felt compelled to come to hospital. For them, every visit was necessary, and dismissal of their needs by staff was interpreted as disrespect and prejudice.  Further training  and sensitization to the needs of individuals in psychiatric  crisis is required for hospital security staff, who are often  involved in volatile interactions with these individuals. |
| Brunero et al. (2007) | Australia | To determine the clinical characteristics of people with mental health problems who frequently attend an Australian emergency department (ED). | Quantitative  Quality score 12 | A retrospective clinical audit of presenter characteristics | N= 868 | 12.5% attended at least twice. The entire frequent presenters’ group had their second presentation within the first month and 10 (77%) had all their presentations within the first 6 months. The occasional repeaters had their second visit to the ED within the first month 65% of the time, and all visits within 6 months. | Younger people appeared more prominently in the frequent presenters group this group also contained more mood/anxiety diagnoses than the other groups. Frequent presenters were more likely to be self-prompted arrivals and less likely to arrive via ambulance or police |
| Spence et al, (2008) | Canada | To investigate the repeated use of the E.D by men with a history of suicidal behaviour and substance abuse to understand the needs and barriers to care. | Qualitative  Quality score Medium | Semi structured interviews, patients, ED staff and family physicians | N=25 patients  N=17  ED staff members | The ED was viewed as a last resort despite seeking help. Frustration was felt by both patients and staff regarding difficult communication, especially during an acute crisis. Of the health care workers interviewed, 77% also stated that these patients had negative experiences.  Staff felt that patient visits were stressful because of their repetitive nature and the difficulty conducting assessments, especially when there were competing demands for their time. | All patients reported that they had previous negative ED experiences that impacted their ability to seek and find ongoing care, especially after being identified as “frequent  flyers.” Recognizing the limitations of the setting, ED staff should attempt to optimize their interactions with these patients. |
| Kuehl et al, (2012) | New Zealand | To describe the number, characteristics and management of patients who  presented to an emergency department (ED) with intentional self-harm and then represented for any reason within 1 week, over a 1-year period. | Quantitative  Quality score 11 | A retrospective records review from one New Zealand ED over 12 months. | N=48 | A group of patients re-presented to ED within days.  following ISH. Of concern was the risk of further serious ISH which was evidenced  by increased inpatient admission numbers. A significant number of patients (54%)  were involved in challenging incidents, demonstrating they were distressed,  experiencing a mental health crisis and possibly were at risk to self and/or others. | While patients with mental health issues often report that general staff have negative  attitudes toward them,17 some doctors have reported feeling helpless in addressing the  emotional aspects of self-harm. A decreased level of consciousness, assumed of some patients post overdose, can also  make a mental health assessment in ED difficult. |
| Joubert et al, (2012) | Australia | To examine the psychosocial precipitating factors of people presenting to the emergency department  (ED) due to attempted suicide. | Quantitative  Quality score 12 | 2 stages- Clinical Data Mining and Action Research | N = 72 | The majority of patients (78%) received their care in the ED, with 12% requiring admission to a ward for management of a medical condition. Of the 72 patients, 68% were discharged to their home, 11% to another  acute hospital, 6% to an inpatient psychiatric facility, and 11% discharged  themselves against the advice of the treating team. | 62% of presentations to the ED occurred outside of  regular business hours. Depression was present in 92% of cases (using Beck’s Depression Inventory). Patients largely do not follow up on referrals  or return to their primary care physician as advised |
| Watson et al, (2008) | U.S.A. | To explore police  encounters from the perspective of persons with mental  illness | Qualitative  Quality score High | Using procedural justice theory as a  sensitizing framework- in-depth semi-structured  interviews | N=20 | Two major themes emerged from our interviews with  people with mental illness. First, the respondents are  fearful of the police and second, the behaviour of the police  during these encounters affects the corresponding experiences and behaviour of the respondents | Participants came into contact with police in a variety of  ways, two main themes emerged. First, they feel vulnerable  and fearful of police, and second, the way police treated  them mattered. |
| Wise-Harris et al. (2017) | Canada | To explore perceived need  for and experiences of ED utilization of  frequent users with mental health and/or addictions challenges | Mixed methods  Quality score 13 | Quantitative  surveys in-depth, qualitative interviews | Quant  surveys (N = 166) and in-depth, Qual  interview  (N= 20) | Participants presented to hospital for mental health (35 %),  alcohol/drug use (21 %), and physical health (39 %) concerns A perceived clash of viewpoints among patients, community service providers, and ED personnel as to the appropriateness of the ED as a point of care for mental  health and substance use crises | Participants described their ED visits as unavoidable and  appropriate, despite feeling stigmatized by hospital personnel and being discharged without expected treatment |
| Clarke et al, (2007) | Canada | Themes identified were waiting in  the ED, attitudes of treatment staff, diagnostic overshadowing, ‘nowhere else to go’, family needs,  and a wish list for ideal services. | Qualitative  Quality score High | Eight focus groups | N=27 clients, n=7 family  members  N=5 stakeholders |  | Many of those currently coming for help  because there is nowhere else to go. Need for safe, respectful, and holistic care that recognises each  client as a worthwhile individual with complex medical  and mental health needs. |
| O’Keefe et al (2021) | England | To explore treatment of self-harm in emergency departments, comparing perspectives of patients, carers and practitioners. | Qualitative  Quality score High | Focus groups and semi-structured interviews | N=79 | Four themes identified:  (a) the wider system is failing people who self-harm (b)practitioners feel powerless and become hardened towards patients, with patients feeling judged for seeking help which exacerbates their distress; (c) patients need a human connection to offer hope when life feels hopeless, yet practitioners underestimate the therapeutic potential of interactions; and (d) practitioners are fearful of blame if someone takes their life: formulaic question-and-answer risk assessments help make staff feel safer but patients feel this is not a valid way of assessing risk or addressing their needs. | Emergency department practitioners should seek to build a human connection and validate patients’ distress, which offers hope when life feels hopeless. Patients consider this a therapeutic intervention in its own right. Investment in self-harm treatment is indicated. |
| Theme 2. Intoxication, self-harm, and aggression | | | | | | | |
| Borges et al, (2006) | International | To study the risk of non-fatal injury at low levels and moderate levels of alcohol consumption as well as the differences  in risk across modes of injury and differences among people with alcohol dependence | Quantitative  Quality score 13 | A case–crossover method to compare the use of  alcohol during the 6 hours prior to the injury with the use of alcohol during same day of the week in the previous week. | 10 E. Ds  around the world (n =4320) | The risk of injury increased with consumption of a single drink (odds ratio (OR) = 3.3; 95% confidence interval = 1.9–5.7),  and there was a 10-fold increase for participants who had consumed six or more drinks during the previous 6 hours. Participants  who had sustained intentional injuries were at a higher risk than participants who had sustained unintentional injuries. Patients who had no symptoms of alcohol dependence had a higher OR | Since low levels of drinking were associated with an increased risk of sustaining a non-fatal injury, and patients who  are not dependent on alcohol may be at higher risk of becoming injured, comprehensive strategies for reducing harm should be  implemented for all drinkers seen in emergency departments |
| Larkin et al, (2017) | Ireland | To identify factors associated with alcohol consumption in cases of suicide and nonfatal self-harm presentations | Quantitative  Quality score 11 | Exploration of the National Self-Harm Registry Ireland | N=5,858 | Between January 2007 and December 2013, there were  8,145 self-harm presentations to EDs  involving 5,858 Alcohol is reported in the case  notes of over one fifth of self-harm presentations. Alcohol involvement was associated with younger age  in suicides and with older age in self-harm presentations. | Alcohol consumption commonly precedes suicidal behavior, and several factors differentiated alcohol-related suicidal acts. Self-harm cases, in particular, differ in profile when alcohol is consumed and may require a tailored clinical approach  to minimize risk of further nonfatal or fatal self-harm |
| Griffin et al, (2017) | International | To establish the role of alcohol in self-harm as well  as to identify associated factors, to best inform service provision | Quantitative  Quality score 14 | Comparative data on hospital-treated self-harm from both the National Self-Harm Registry Ireland and the Northern Ireland  Registry of Self-Harm. | N=24 513 | 19 831 (58%) were made to EDs in Ireland and 14 598 (42%)  to EDs in Northern Ireland. Approximately 52% of the sample were female and 44% were aged between 25 and 44 years.  The most common method of self-harm recorded was intentional drug overdose (71%). The only other common method  of self-harm was self-cutting, present in 23% of acts. Fewer  than one-third (29%) were repeat presentations and 31%  were made by residents of urban areas. | Alcohol was present in 43% of all self-harm acts, and more common in Northern Ireland (50 versus 37%). The factors associated with  alcohol being involved were being male, aged between 25 and 64 years, and having engaged in a drug overdose or attempted drowning.  Presentations made out-of-hours were more likely to have alcohol present and this was more pronounced for females. Patients with alcohol on  board were also more likely to leave without having been seen by a clinician |
| NHS Quality improvement Scotland (2008) | Scotland | Audit into harmful drinking | Quantitative  Quality score  11 | Audit of mainland E. D’s | N=15 | More than half of those presenting with self-harm injuries had consumed alcohol prior to attending emergency services. | 27% of men and 19% of women cited alcohol was a trigger for self-harming, supporting evidence of alcohol consumption and intoxication as a key co-occurring factor in the management of PiMD presenting through emergency services |
| Downes, et al, 2009) | Australia | To describe the characteristics of patients with acute behavioural disturbance and their  emergent treatment in an ED with a structured team approach | Quantitative  Quality score 12 | Retrospective review of acute behavioural emergencies that required response  from the Code Black (CB) Team (duress response team) in the ED | N=122 | 71 male patients (58%) who accounted for 143 CB activations. The primary problems  were deliberate self-poisoning or self-harm (38%), alcohol and illicit drug intoxication (33%)  and psychiatric, organic illness and drug withdrawal (29%). One hundred and eight (89%)  patients had a history of alcohol/illicit drug abuse or psychiatric illness. Indications for  CB activations were threatening harm to others or behaving violently in 67% of cases | Acute behavioural disturbance is a common occurrence  in ED, the frequency of these events is  signiﬁcantly higher than previously quoted in the  Australasian ED literature. Underlying causes were  predominantly organic in nature, and a team approach  appears to be invaluable in managing these incidents |
| Zisman & O’Brien, (2015) | Australia | To identify the demographic profiles, circumstances of detention and assessment outcomes of all individuals detained under Section 136. The study explored the relationship between alcohol and/or drug use, the process and outcomes. | Quantitative  Quality score 12 | This retrospective cohort study | N=245 | Threatening to self-harm (n = 100, 44.8%) was the most common reason for assessment. Of the 245 patients assessed, 108 (44.1%) were found to be intoxicated with drugs and/or alcohol. | Intoxication resulted in longer assessment times and a decreased likelihood of admission to hospital (p < .000). |
| Maharaj, et al, (2011) | Australia | To identify any differences between patients referred by police compared with patients referred from other sources to a psychiatric hospital in Australia | Quantitative  Quality score 13 | retrospective audit | N=200 | The two most common reasons for the involuntary referral of patients by police were bizarre ideas (33%) and threats of suicide (28%). When 101 patients referred by police were compared with 99 patients from other sources, police referrals were three times more likely to be diagnosed with a mental and behavioural disorder because of psychoactive substance use, less likely to be diagnosed with a mood disorder, and less likely to be diagnosed as psychotic. | Police referrals were more likely to have worse functional scores; exhibit aggressive behaviour; spend fewer days in hospital; more likely to be admitted to the psychiatric intensive care unit, and to be secluded. The most important predictor for a police referral was drug or alcohol problems. |
| Morphet et al, (2014) | Australia | To identify the causes and common acts of violence in the ED perceived by three distinct groups of nurses. | Quantitative Quality score  12 | Delphi technique | Round 1 n=157  Round 2 n= 132  Round 3 n=158 | Long waiting times, drugs and alcohol all contributed to ED violence | Triage nurses indicated that ED staff, including security staff and the triage nurses themselves, can contribute to violence |
| Doyle et al, (2007) | Ireland | To describe the experiences and  challenges that nurses encounter when caring for patients who present  to the ED with suicidal behaviour. | Qualitative  Quality score Medium | 15-item semi-structured questionnaire | Forty-two ED nurses | Participants in this study  identified risk assessment as part of their role but did not focus on psychosocial  assessment or psychological management of this  patient group. Feelings of sympathy and compassion were reported  towards patients, there was often a prior judgement  of the perceived ‘genuineness’ of the presentation. | Challenges experienced included a lack of appropriate communication skills and insufficient resources within the ED to adequately care for this  vulnerable patient group. |
| Lord & Bjerregaard (2014) | U.S.A. | To determine (1) the nature of  interactions between police-referred calls to the mental health mobile crisis unit and (2) to determine the outcomes  of these calls and the factors that might influence the outcomes. | Quantitative  Quality score 12 | Comparison between law enforcement–initiated calls and those initiated by other  agencies/individuals | N= 3,635 | Significant differences  in the type of PMI calls from law enforcement–referred calls to MCUs are found; the  PMI are twice as likely to be violent, intoxicated, psychotic, mood-order diagnosed, and  in emergent need of care | Law enforcement–referral calls primarily deal with persons with serious mental illness (PSMI) and who are in crisis. Without appropriate and immediate mental health intervention, law enforcement officers might have to resort to physical restraint and arrest |
| Maharaj, et al, (2013) | Australia | Explore nurses' experience of caring for police‐referred patients to psychiatric hospitals | Qualitative  Quality score Medium | Semi structured interviews | N=9 | The theme ‘Expecting the worst’ was constituted by the sub‐themes of: (i) ‘we are here to care for whoever they bring in’; and (ii) ‘but who deserves care?’ The theme balancing therapeutic care and forced treatment was constituted by the sub‐themes of: (i) ‘taking control, taking care’; and (ii) ‘managing power’. | Ethical issues related to stereotyping of patients brought in by police and labelling as ‘the worst’ patients, and what this may mean in terms of subsequent treatment. A lack of operationalized models of care and clinical guidelines for managing the range of patients including those with dual diagnosis. A lack of resourcing and expertise, and failure of continuity of care and collaboration between specialist services such as substance use services |
| Theme 3. Professional perspectives and responses to PiMD | | | | | | | |
| McAllister et al, (2002) | Australia | To develop and test a scale to identify relevant dimensions of ED nurses' attitudes to clients who present with self‐injury. | Quantitative  Quality score 12 | Questionnaire  (ADSHQ) were drawn from a literature review and focus group discussions with ED  nurses. The tool was piloted with 20 ED nurses not working in the target agencies. | N=352 | Four factors that reﬂected nurses’ attitudes toward these clients were related to; nurses’ perceived conﬁdence in their assessment and referral skills; ability  to deal effectively with clients; empathic approach; and ability to cope effectively  with legal and hospital regulations that guide practice | There was a generally negative attitude towards clients who self-harm. Correlations were found between years  of ED experience and total score on the ADSHQ, and years of ED experience and an  empathic approach towards clients who deliberately self-harm. |
| Summers & Happell, (2003) | Australia | To ascertain the level of psychiatric patient satisfaction with the services received in the emergency department of a Melbourne metropolitan hospital. | Qualitative  Quality score High | Telephone interview. A structured questionnaire was used to seek information from patients regarding what was helpful, and what was not helpful, about their treatment, and sought any feedback that could improve the service. | N=136 | High level of satisfaction, particularly with the availability of staff with psychiatric qualifications and experience to provident treatment, support and care. The major areas of dissatisfaction identified by patients included: lengthy waiting times, lack of privacy in the triage area and negative attitudes of general staff. | Need for triage guidelines to be tailored to the needs of mental health patients and for emergency department triage staff to be appropriately educated to adequately triage these patients. |
| Conlon & O’Tuathail, (2012) | Ireland | to measure emergency department nurses’ attitudes towards  deliberate self-harm. | Quantitative  Quality score 12 | The ‘Self-Harm Antipathy Scale’, a validated questionnaire, was administered to a random sample of nurses in four emergency departments | N=87 | Nurses show  slightly negative antipathy overall, indicating positive attitudes towards self-harming patients.  Attitudes were significantly different in accordance with a nurse’s age. Education and social  judgment also contribute to the way nurses view, interact and make moral decisions regarding  self-harm patients | Judgmental  attitudes and manipulation in the self-harming behaviour.  Nurses were frustrated with patients frequently  returning to hospital. Moreover, the results of this study  show that the nurses do not feel adequately trained to respond, and many have doubts about the degree of support  that may be available to them in this area. They also feel  that self-harm is the responsibility of the mental health services, and consequently it will indeed be difficult to shift  the major responsibility for responding to self-harm patients to generic hospital-based staff |
| Chapman & Martin, (2014) | Australia | Explore staff perceptions about caring for patients who present to the emergency department following deliberate self-poisoning | Qualitative  Quality score  Medium | Two open-ended questions | N=186 | Three themes emerged from the data representing staff perceptions about caring for patients who deliberately self-poisoned and included depends on the patient, treat everyone the same, and skilled and confident to manage these patients. | Staff reported mixed reactions to patients presenting with deliberate self-poisoning. These included feelings of empathy or frustration, and many lacked the skills and confidence to effectively manage these patients. |
| Commons Treloar & Lewis, (2008) | Australia | To assess the attitudes of mental health and  emergency medicine clinicians towards patients diagnosed with borderline personality  disorder. T | Quantitative  Quality score 13 | Questionnaire | N=140 | Statistically and clinically significant differences were found between emergency  medical staff and mental health clinicians in their attitudes towards working with borderline  personality disorder. The strongest predictor of attitudes was whether the clinician worked  in emergency medicine or mental health. This was followed by years of experience and  specific training in personality disorders as significant predictors of attitudes to self-harm | Mental health clinicians who  provide ongoing management and treatment for  BPD have more positive attitudes to working with  this patient group, and this may be influenced by  their more sustained level of care. Emergency  medicine clinicians are required to provide urgent  medical attention to the patient with BPD following  episodes of self-harm and this may result in such  professionals having a greater difficulty in maintaining an empathetic attitude to such patients |
| Betz et al, (2013) | U.S.A. | To examine the knowledge, attitudes, and practices  of emergency department (ED) providers concerning suicidal patient care and  to identify characteristics associated with screening for suicidal ideation (SI). | Quantitative  Quality score 12 | Survey | N=631 | Providers expressed  scepticism about the preventability of suicide, despite evidence  that suicide prevention measures can prevent deaths. A minority of providers reported screening most or all patients for suicidal ideation | Providers expressed  gaps in their skills and practices related to risk assessment and provision of referral resources Individual beliefs can also be a barrier to care of suicidal patients |
| McCann et al, (2006) | Australia | To assess if accident and emergency (A&E) nurses attitudes towards patients with deliberate self-harm, and to assess if nurses’ age, length of A&E experience, or in-service education influence their attitudes towards these patients. | Qualitative  Quality score medium | Questionnaire | N=43 | Most nurses had received no educational preparation to care for patients with self-harm. Over 20% claimed the department either had no practice guidelines for DSH or they did not know of their existence; one-third who knew about them had not read them | Older and more experienced nurses had more supportive attitudes than younger and less experienced nurses. Nurses who had attended in-service education on DSH had more positive attitudes than non-attendees. |
| Friedman et al, (2006) | England | To investigate the attitudes of accident and emergency (A&E) staff towards patients who self-harm through laceration. | Qualitative  Quality score medium | questionnaire | N=117 | The staff held a belief that self-laceration was an important problem but felt unskilled in managing patients. Evidence of unhelpful attitudes amongst some staff. This is particularly true for more senior staff without previous DSH training, who, as a group, were less sympathetic to this group of patients. | Staff without previous training, a longer period working in A&E was correlated with higher levels of anger towards patients and an inclination not to view patients as mentally ill. A&E staff were keen for further training and wanted a higher proportion of patients to be seen by specialist mental health services. |
| Thompson et al, (2008) | England | To explore community psychiatric nurses' experiences of working with people who self‐harm | Qualitative  Quality score medium | Semi-structured interview | N=8 | Participants described struggling to conceptualize self‐harm behaviour and generally reported finding working with people who self‐harm stressful particularly in terms of managing the emotional impact upon themselves and the boundaries of their professional responsibilities in relation to managing risk | The therapeutic relationship was viewed as crucial and a variety of coping methods to manage the impact of the work, which had largely developed through ‘on the job’, experience were described. |
| Martin & Thomas  (2015) | Australia | To examine police  encounters with people experiencing mental disorder | Qualitative  Quality score high | Semi-structured interviews | N=25 | Ofﬁcers singled out people with personality disorder and expressed frustration, anger, powerlessness and resignation with their referrals of this group to health services. Ofﬁcers  reported that E. Ds were reluctant to assess people with P.D and when they did assess them stated that the person did not meet criteria for admission to mental health services, or if admitted, they were quickly discharged | People with personality disorder were reported to take up considerable police  resources. When police were told by mental health professionals that there was  nothing they could do about people experiencing personality disorder, then the  question from police was what was to be done with them |
| Godfredson et al, (2011) | Australia | To explore (1) the frequency of contact between the police and  people experiencing mental illness; (2) the way in which police officers’ knowledge and the  sources of information used relates to various dispositions; (3) the signs, symptoms and  behaviours that police officers consider are associated with mental illness; and (4) the challenges police face in this respect when performing their duties | Qualitative  Quality score High | Survey | N=3,534 | Police reported that a considerable amount of their time each week was  spent dealing with people they believed to be mentally ill. These encounters were reportedly  associated with considerable practical difficulties for police, both in terms of knowing how to  deal with people experiencing mental illness and how to best find appropriate supports for  them | The most common results of their encounters were instigating a mental health apprehension, followed by arrest, but decision-making was influenced by the differential weight  police placed on different sources of information received at the scene. |
| Mclean & Marshall (2010) | Scotland | To investigate police officers’ views on their roles in dealings with people  with mental health problems and with mental health services. | Qualitative  Quality score  Medium | Semi-structured interviews | N=9 | Recurrent themes identified were: Emotional aspects of dealing with  people with mental health problems and with services, impact of incidents on police  resources and on people with mental health problems, success through collaborative  working with health services and failure in its absence. | police officers interviewed expressed compassion and understanding of  people with mental disorders. Difficulties experienced when called to resolve situations involving the mentally ill, there may be no indication for arrest, but they may be unsuccessful in  securing care or hospital admission for the individual |
| Al-Khafaji et al, (2014) | Australia | To describe the characteristics and outcome of patients brought to an emergency department by police under Section 10 of Mental Health Act | Quantitative  Quality score 13 | Retrospective medical record review | N= 164 | Patients were predominantly male (58%) with median age of 35 years. The most common presenting  complaint (65%) was threat of self-harm. No sedation or restraint was used in 61%. Sixty seven percent were  deemed safe for discharge home while 26% were admitted to a psychiatric ward (equally divided between  voluntary and involuntary admission). The predominant discharge diagnosis was self-harm ideation or intent  (35%). Median ED length of stay was 156 min (inter-quartile range 79–416). | Most patients brought to ED by police under Section 10 provisions were for threat of self-harm and  did not require sedation or restraint. The majority are discharged home. Further work exploring less restrictive  or traumatic processes to facilitate psychiatric assessment of this group of patients is warranted. |
| Rees et al, 2016 | England | to explore paramedics' perceptions and experiences  of caring for people who self-harm | Qualitative  Quality score high | Semi-structured interviews | N=11 | Two emerging themes: Firstly, professional, legal,  clinical and ethical tensions, linked to limited decision support, referral options and education. The second  theme of relationships with police, revealed practices and surreptitious strategies related to care and detention,  aimed at overcoming complexities of care | Paramedics van be conflicted by clinical, legal and ethical care of people who self-harm. To facilitate attendance at hospital paramedics can coerce and collude with police. Whilst this may appear distasteful, dishonest, unprofessional, unethical or illegal, conditions that cultivate  such practices need to be questioned. |
| Fry et al, (2002) | Australia | To examine the relationship between mental health services and police and people who  have mental health problems, are suicidal or are drug- and/or alcohol affected | Qualitative  Quality score High | Survey | N=131 | More than 10% of police time is spent dealing with  people with mental health problems. Nevertheless, police felt unsupported  in this role, unprepared for it and torn between the competing demands  experienced in their work. A lack of confidence in dealing with suicidal  people and a belief that work with mentally disturbed people does not  constitute valid police work | There are difficulties relating to: inadequate training and education; deficiencies in  services/resources; time and resource over-utilization; communication,  liaison and feedback problems, and frustration related to accessing mental  health facilities/services. It is argued that police work involving mentally  disturbed people are a valid and necessary role that complements law  enforcement |
| Schulenberg  (2016) | Canada | To explore the ways in which the decision-making process and use  of discretion with people with mental illness (PMI) reflects a systematic procedural bias that can directly or indirectly  contribute to criminalization of the mentally ill. | Mixed methods  Quality score 12 | Observational data analysed (1) binary variables were calculated and the  chi-square statistic used to assess whether the differences between PMI and non-PMI were statistically significant. (2) Using  three binary logistic regression models  and (3) thematic analysis | Data from 637 hrs of ‘ride- alongs’ with police officers | Regardless of mental health status, a citizen who is under the influence of  alcohol or drugs increases the likelihood of criminal charges and citations. Evidence of indirect and direct pressure to resolve PMI-related calls in an expedient  manner. | Patterns in police response strategies, including behavioural indicators  officers use as criteria for determining mental health status, procedural challenges with  limited resources or information, and complications endemic to PMI encounters that culminate in constraints on officers’ decision-making autonomy. |
| Cotton (2004) | Canada | To identify and quantify those attitudes that may influence the  discretionary behaviour of police in their interactions with people with people they believed to mental illness | Quantitative  Quality score 13 | Questionnaire | N=138 | Police officers are in the untenable position of  having a social expectation to ‘‘do something,’’ while at the same time, having no clear reason to arrest  and knowing full well that a visit to the local emergency room is unlikely to lead to admission or  treatment, unless the individual in question is acutely homicidal or suicidal. | Police officers appear to  have attitudes similar with those of the public, and do not ascribe to punitive  and isolationist attitudes that might lead to the apprehension of mentally ill individuals without a clear  reason for such action. They are interested in obtaining more information about working with  and understanding individuals who are mentally ill. |
| Van Den Brink et al, (2012) | The Netherlands | To examine the extent to which these individuals are disconnected from mental health services, and  whether the police response has an influence on re-establishing contact | Qualitative  Quality score  High | Retrospective review of police records | N=336 | Half of people with mental health needs coming to police attention (N=162) were disengaged from mental health services, lacking regular care contact in the year prior to the crisis  In the month following the crisis, 21% of those who were previously disengaged from services had  regular care contact, and this was more frequent (49%) if the police had contacted the mental health services  during the crisis. The  majority (58%) of disengaged individuals’ police did not contact the mental health services at the time of crisis. | Police play an important role in linking these individuals  to services. There may be room for improvement, as more  than half of the individuals who were disengaged from  mental health services were not connected with services  at the time of crisis. |
| Watson et al (2021) | U.S.A | Examine the impact of CIT response on outcomes of mental health-related police encounters. | Quantitative  Quality score 14 | Surveys focused on police encounters on mental -health related calls | N=428 | The only significant predictor of the call resulting in arrest rather than other outcomes is whether the call was pre-identified as a mental health call. CIT response increases the odds that a call will result in linkage to mental health service. Location of call and pre-identified mental health call are both statistically significant, but CIT response is not. Calls that originate from a street or park—or from a business, government building, police station, or transit—are more likely to have an informal resolution than calls that originate from a home | Multiple factors may shape the outcomes of mental health-related encounters with police. CIT response, dispatch coding, and the places where calls originate play a role in shaping outcomes. Whether or not calls were identified during the call-taking and dispatch process as mental health related had a significant effect on how calls are resolved. Officers were more likely to link people to services, including EDs, if they knew in advance the calls had a mental health component. |
| Wood et al (2021) | U.S A | Exploration of perspectives on the unmet needs of individuals and their families and the ways in which the mental health and social system environment constrain officers' abilities to be responsive to them. | Qualitative  Quality score High | qualitative data gathered from field observations | N=36 | Officers responded to a variety of mental health-related calls revealing complex, unmet needs at individual and family levels. A common theme related to officers' perceptions that “going off meds,” combined with other situational factors, resulted in police being involved in behavioural health situations. The data also revealed broader aspects of the health and social system that, in officers' minds, constrain their ability to effect positive outcomes for people and their families, especially in the long term. | Findings underscore the need for cities and communities to develop alternatives to emergency departments which, in the long term, may provide the best hope for reducing the reliance on police as mental health interventionists. Formal collaborations between the law enforcement community and the mental health nursing community could be focused towards this end. |
| Lamb, V, and Tarpey, E. (2019). | England | Explore police officers’ views of their experience of working with people with mental health difficulties and their experience of training to equip them for this | Qualitative  Quality score  High | Semi structured interviews - police officers in one force in England | N=10 | Three key themes emerged from the interviews with the officers regarding their experiences of working with people with mental health difficulties: Support, Impact on the Officer and Understanding Mental Health. | Conflict and confusion are being caused between agencies where mental health is involved. The introduction of Street Triage Team and the police-health nexus have gone some way to resolving this confusion/conflict, appearing to increase officer confidence in recognition and knowledge of mental health, whilst reducing officer workload. However, access to the Street Triage Team is limited and workload/multi-agency issues remain. |
